# The activity of the RGA5 sensor NLR from rice requires binding of its integrated HMA domain to effectors but not HMA domain self‐interaction

**DOI:** 10.1111/mpp.13236

**Published:** 2022-06-29

**Authors:** Yuxuan Xi, Véronique Chalvon, André Padilla, Stella Cesari, Thomas Kroj

**Affiliations:** ^1^ PHIM Plant Health Institute, Univ Montpellier, INRAE, CIRAD, Institut Agro, IRD Montpellier France; ^2^ CBS, Univ Montpellier, CNRS, INSERM Montpellier France

**Keywords:** disease resistance, effector, heavy metal‐associated domain, immune receptor, *Magnaporthe oryzae*, NLR, rice

## Abstract

The rice nucleotide‐binding (NB) and leucine‐rich repeat (LRR) domain immune receptors (NLRs) RGA4 and RGA5 form a helper NLR/sensor NLR (hNLR/sNLR) pair that specifically recognizes the effectors AVR‐Pia and AVR1‐CO39 from the blast fungus *Magnaporthe oryzae.* While RGA4 contains only canonical NLR domains, RGA5 has an additional unconventional heavy metal‐associated (HMA) domain integrated after its LRR domain. This RGA5_HMA_ domain binds the effectors and is crucial for their recognition. Investigation of the three‐dimensional structure of the AVR1‐CO39/RGA5_HMA_ complex by X‐ray crystallography identified a candidate surface for effector binding in the HMA domain and showed that the HMA domain self‐interacts in the absence of effector through the same surface. Here, we investigated the relevance of this HMA homodimerization for RGA5 function and the role of the RGA5_HMA_ effector‐binding and self‐interaction surface in effector recognition. By analysing structure‐informed point mutations in the RGA5_HMA_‐binding surface in protein interaction studies and in *Nicotiana benthamiana* cell death assays, we found that HMA self‐interaction does not contribute to RGA5 function. However, the effector‐binding surface of RGA5_HMA_ identified by X‐ray crystallography is crucial for both in vitro and in vivo effector binding as well as effector recognition. These results support the current hypothesis that noncanonical integrated domains of NLRs act primarily as effector traps and deepen our understanding of the sNLRs' function within NLR pairs.

## Graphical abstract

Mutant analysis validates the effector‐binding surface in the noncanonical integrated HMA domain of the rice immune receptor RGA5 and shows that its self‐interaction does not contribute to RGA5 function.

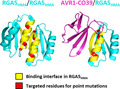

## 1 INTRODUCTION

Plants rely on two types of immune receptors to recognize pathogen invasion: cell surface receptors that detect extracellular invasion patterns and intracellular receptors that recognize pathogen‐secreted effectors acting inside the host cell (Cook et al., 2015). Intracellular plant immune receptors belong to the family of nucleotide‐binding and leucine‐rich repeat domain proteins (NLRs). NLRs are multidomain proteins characterized by a conserved central NB‐ARC (nucleotide‐binding adaptor shared by Apaf1, certain R genes and CED4 family proteins) domain that acts as a molecular switch and mediates NLR activation upon ligand recognition. In addition, NLRs carry a C‐terminal leucine‐rich repeat (LRR) domain that is crucial for signal perception and a variable N‐terminal domain that activates downstream signalling (Maekawa et al., 2011). Most commonly, this N‐terminal signalling domain is either a CC (coiled‐coil) or a TIR (Toll‐interleukin 1 receptor) domain allowing the classification of most NLRs into either CNLs or TNLs (Shao et al., 2016). In addition to these canonical domains, some NLRs also carry highly variable and flexibly located unconventional integrated domains (ID) and are therefore called NLR‐IDs (Bailey et al., 2018; Cesari, Bernoux, et al., 2014; Kroj et al., 2016; Sarris et al., 2016).

Structure–function analysis revealed that effector recognition by plant NLRs triggers their aggregation in multimeric complexes named resistosomes (Ma et al., 2020; Martin et al., 2020; Wang, Hu, et al., 2019; Wang, Wang, et al., 2019). This multimerization is mediated in large part by the NB‐ARC domain and involves, in addition, the N‐terminal CC or TIR domain. The effector‐triggered assembly of the CNL ZAR1 into pentameric resistosomes results in the formation of a calcium‐permeable ion channel that integrates into the plasma membrane and initiates immune responses (Bi et al., 2021; Wang, Hu, et al., 2019; Wang, Wang, et al., 2019). The TNLs ROQ1 and RPP1 assemble into tetrameric resistosomes, where the NADase activity of their TIR domains is activated (Ma et al., 2020; Martin et al., 2020).

Frequently, the recognition of effectors by NLRs is not direct, that is, by physical binding of the ligand to the receptor, but occurs in an indirect manner and involves either host proteins targeted by the effector or mimics of effector targets that are called decoys (van der Hoorn & Kamoun, 2008). IDs of NLR‐IDs are supposed to act as integrated decoy domains, although this has only been demonstrated in rare cases (Cesari, Bernoux, et al., 2014; Le Roux et al., 2015; Maqbool et al., 2015; Ortiz et al., 2017; Sarris et al., 2015). While some NLRs act alone and combine the functions of effector sensing and activation of downstream immune signalling, others work in pairs composed of one sensor NLR (sNLR) and one helper (or executor) NLR (Adachi et al., 2019; Feehan et al., 2020). NLR‐IDs work systematically in pairs where they act as sNLRs.

Blast disease caused by *Magnaporthe oryzae* is one of the most severe diseases of rice, provoking important losses, estimated at 4% of the global production, and threatening the stable supply of this outstandingly important staple crop (Savary et al., 2019). The genetic resistance of rice to blast largely relies on NLRs. Their efficiency is challenged by the tremendous adaptive potential of *M. oryzae*, which allowed the fungus to rapidly overcome numerous resistance (*R*) genes in the past (Wang et al., 2017). A better understanding of the mechanisms of effector recognition and immune activation by NLRs is therefore essential for a more sustainable protection of rice against blast.

NLR pairs composed of one helper NLR and one NLR‐ID acting as an sNLR are particularly frequent among rice R proteins conferring resistance to blast. A model system for their understanding are the CNLs RGA4 and RGA5, which are both required for the specific recognition of the *M. oryzae* effectors AVR‐Pia and AVR1‐CO39 (Cesari et al., 2013; Okuyama et al., 2011). Both these effectors belong to the MAX effector family characterized by similar three‐dimensional structures but completely different amino acid sequences (de Guillen et al., 2015). The helper NLR RGA4 is an auto‐active inducer of immune signalling, which is repressed by RGA5 in the absence of effectors (Cesari, Kanzaki, et al., 2014). The sNLR RGA5 carries a heavy metal‐associated (HMA) ID after its LRR domain and recognizes AVR1‐CO39 and AVR‐Pia through direct binding, which relieves RGA4 repression (Cesari, Kanzaki, et al., 2014; Ortiz et al., 2017). This functional sNLR/eNLR interaction involves physical binding because RGA4/RGA5 complexes are formed in both resting and activated states (Cesari, Kanzaki, et al., 2014).

The HMA domain plays a crucial role in the recognition of effectors by RGA5. It binds directly to AVR‐Pia and AVR1‐CO39, and an HMA deletion mutant is insensitive to effectors but still represses RGA4 (Cesari et al., 2013; Cesari, Kanzaki, et al., 2014). The molecular mechanism of the binding of AVR‐Pia and AVR1‐CO39 to RGA5_HMA_ has been elucidated in much detail by structure–function analyses involving nuclear magnetic resonance (NMR) and X‐ray crystallography (Guo et al., 2018; Ortiz et al., 2017). They revealed that both effectors bind RGA5_HMA_ through very similar interfaces mainly formed of β‐strand 2 and residues from the N‐terminus of the MAX effectors located before and at the beginning of β‐strand 1. The crystal structure of AVR1‐CO39/RGA5_HMA_ suggests that the binding interface of RGA5_HMA_ is formed of α‐helix A and β‐strand 2, and modelling suggests that a nearly identical surface mediates AVR‐Pia‐binding (Cesari et al., 2022; Guo et al., 2018). The binding interfaces in the effectors have been confirmed by the analysis of effector mutants in which key residues for HMA binding were replaced. However, the effector‐binding surface of RGA5_HMA_ has not yet been validated experimentally. In addition to RGA5_HMA_ binding, AVR‐Pia associates with additional sites in RGA5, outside of the HMA domain (Cesari et al., 2022; Ortiz et al., 2017). However, details of these interactions and their contribution to effector recognition are not known.

The isolated RGA5_HMA_ domain forms homodimers in vitro and in vivo (Guo et al., 2018). Interestingly, this self‐interaction is established through the same surface that also mediates effector‐binding and it is disrupted in the presence of AVR1‐CO39 or AVR‐Pia (Guo et al., 2018). However, the biological role of RGA5_HMA_ self‐interaction, and whether it occurs in full‐length RGA4/RGA5 complexes, is not clear. It is probably not relevant for the activated state because it is out‐competed by effector binding. However, RGA5_HMA_ could play a role in the proper repression of RGA4 in the absence of effectors.

In this study, we further elucidated the molecular mechanisms of RGA5 function by studying effector binding and self‐interaction of RGA5_HMA_. Using structure‐guided mutagenesis, we found that RGA5_HMA_ dimer formation is not required for RGA5 function. However, the effector‐binding surface of RGA5_HMA_ identified by X‐ray crystallography is crucial for both in vitro and in vivo effector binding as well as effector recognition. This confirms the current hypothesis that IDs of NLRs act primarily as effector traps and deepens our understanding of sNLRs' function.

## 2 RESULTS

### 2.1 Structure‐informed design of RGA5_HMA_ mutations

**FIGURE 1:**
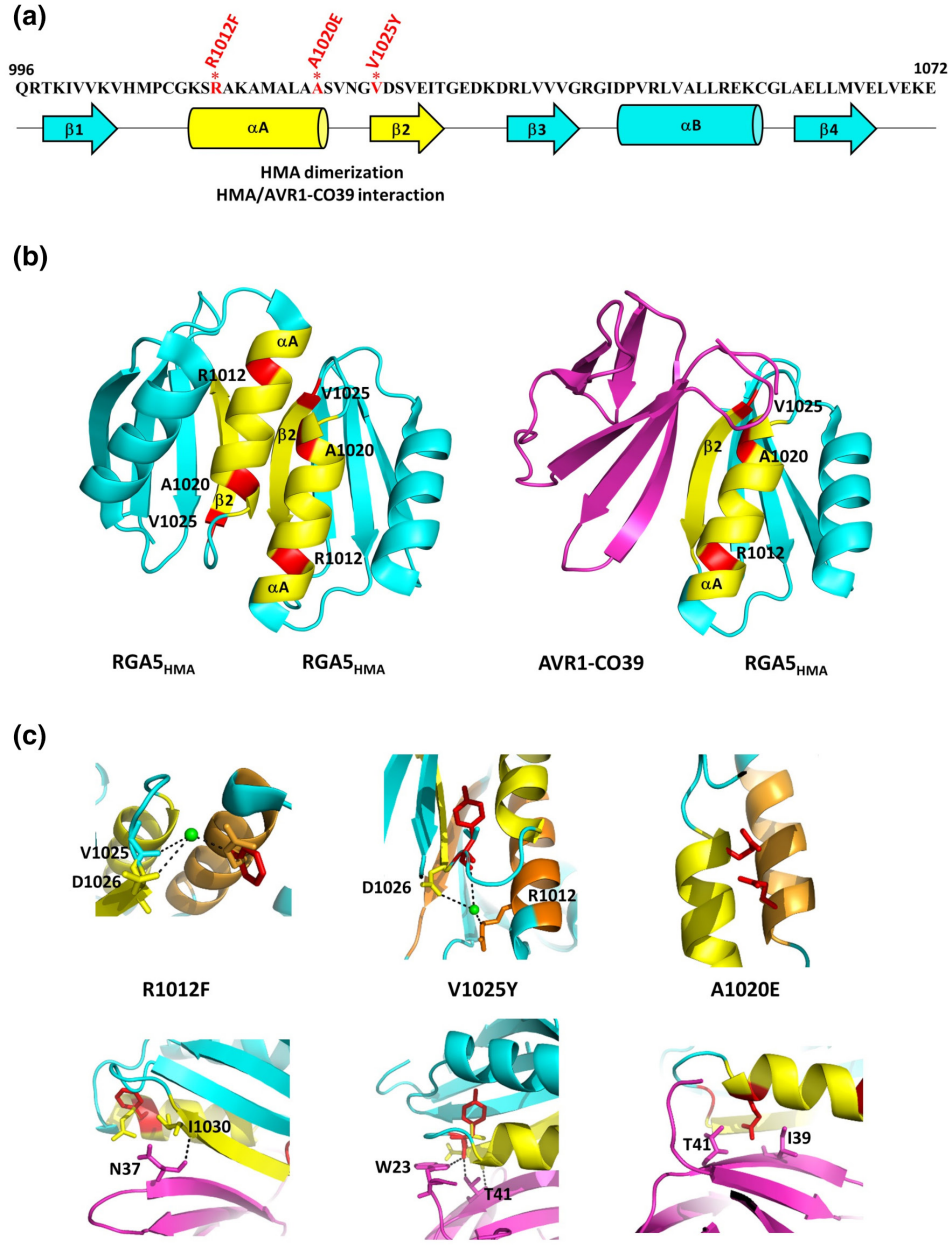
Structure‐informed mutations introduced in the self‐association and effector‐binding surface of RGA5_HMA_. (a) Amino acid sequence and secondary structure of the RGA5_HMA_ domain. Red asterisks indicate the mutated residues and corresponding mutations are marked in red. The self‐association and effector‐binding surface of RGA5_HMA_ (α‐helix A and β‐strand 2) is indicated in yellow. (b) Crystal structures of the RGA5_HMA_ homodimer (left) and the RGA5_HMA_/AVR1‐CO39 complex (right). The αA‐β2 surface of RGA5_HMA_ is highlighted in yellow and the residues selected for targeted substitutions are shown in red. The structure of AVR1‐CO39 (without signal peptide) is shown in purple. (c) Enlarged views of the R1012F, V1025Y, and A1020E mutated residues (red‐coloured side chains). The wild‐type residue side chains are in cyan, yellow, or orange (for the second monomer in the dimer). The top panels show the homodimer with one HMA monomer in yellow and the other in orange, the bottom panels show the heterocomplex with AVR1‐CO39 in purple.

To analyse the role of RGA5_HMA_ homodimer formation in the regulation of RGA4 and in the recognition of AVR1‐CO39 and AVR‐Pia, targeted point mutations were introduced in RGA5_HMA_. We used the FoldX 4 toolkit (Guerois et al., 2002; Schymkowitz et al., 2005) to predict, within the RGA5_HMA_ dimer and the RGA5_HMA_/AVR1‐CO39 complex, the changes in binding energy associated with the individual substitution of key residues of the RGA5_HMA_ interaction surface (Tables S1 and S2). Multiple substitutions in α‐helix A and β‐strand 2 were predicted to strongly perturb both complexes (Tables S1 and S2). Among them, we selected three substitutions of surface‐exposed residues thought to alter both the dimerization and the interaction with AVR1‐CO39, with limited risk to perturb protein structure: R1012F, A1020E, and V1025Y (Figure 1 and Table S3).

In the dimer, R1012 and V1025 contribute to binding by establishing a water bridge with D1026 (Figure 1c). This contact is expected to be destabilized in the R1012F and the V1025Y mutants. In the heterocomplex, V1025 engages together with D1026 a hydrogen bonding network with the AVR1‐CO39 residues W23 and T41 (Figure 1c). This contact, which contributes significantly to RGA5_HMA_/AVR1‐CO39 binding, is destabilized in the V1025Y mutant. R1012 does not engage interactions in the heterocomplex but we predict that its replacement generates a Van der Waals clash with I1030 that forms a hydrogen bond with N37 of AVR1‐CO39. A1020 does not establish interactions in the dimer or with AVR1‐CO39. However, its substitution by a glutamate was anticipated to cause a strong negative charge repulsion of the two glutamates in the dimer and to add penalization for burying the polar glutamate side chain between AVR1‐CO39 residues I39 and T41 in the heterocomplex (Figure 1c). These mutations were introduced into the HMA domain of RGA5 individually or in combination (AE/VY, RF/AE, RF/VY, and RF/AE/VY).

### 2.2 RGA5_HMA_ homodimer formation is impaired by mutations in its self‐interaction and effector‐binding surface

**FIGURE 2:**
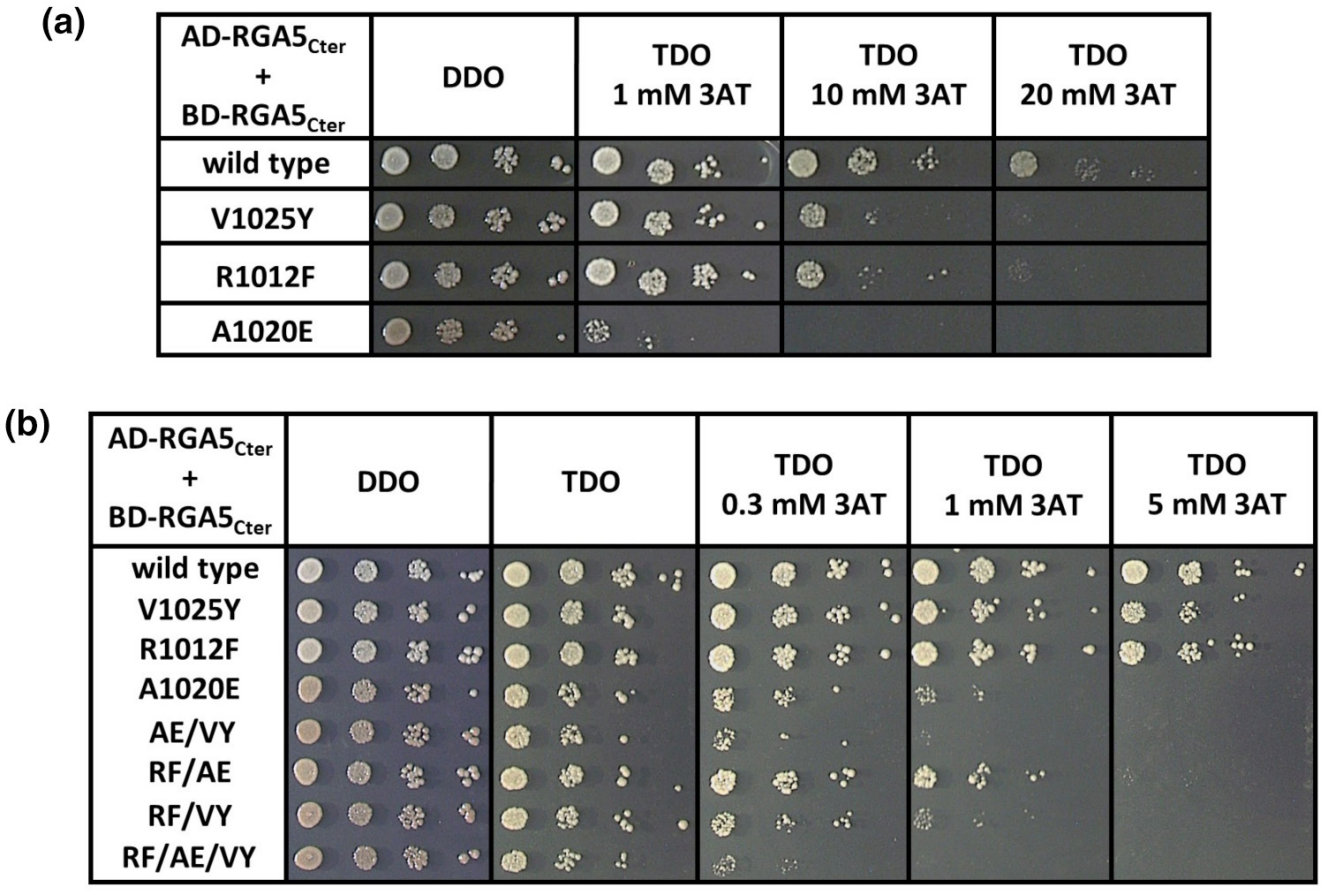
Mutations in the binding surface of RGA5_HMA_ affect RGA5_Cter_ self‐interaction in yeast two‐hybrid assays. (a) Dilution series of diploid yeast cells expressing RGA5_Cter_ constructs (wild type and single mutants) fused to the DNA‐binding domain (BD) and to the activation domain (AD) of the GAL4 transcription factor were spotted on synthetic triple dropout (TDO) medium (−Trp/−Leu/−His) supplemented with 1, 10, and 20 mM 3‐amino‐1,2,4‐triazole (3AT) to assay for self‐interactions. Diluted yeast cells were also spotted on synthetic double dropout (DDO) medium (−Trp/−Leu) to monitor proper growth. Pictures were taken after 5 days of growth. (b) Self‐interaction of RGA5_Cter_ wild type, single, double, and triple point mutants was assayed as in (a) but on TDO supplemented with 0.3, 1, and 5 mM 3AT. Both experiments were performed twice with identical results.

To determine whether the selected substitutions affect RGA5_HMA_ homodimer formation, they were introduced in the C‐terminal fragment of RGA5 (residues 883 to 1116) containing the HMA domain and the self‐interaction of these RGA5_Cter_ variants was examined by yeast two‐hybrid (Y2H) assay. We selected this fragment as it has in vitro and in coimmunoprecipitation (co‐IP) assays the same AVR1‐CO39 and AVR‐Pia‐binding characteristics as the isolated RGA5_HMA_ domain and shows Y2H interaction with effectors and with itself while RGA5_HMA_ does not (Guo et al., 2018; Ortiz et al., 2017). All proteins fused to the Gal4 activation domain (AD) or binding domain (BD) were properly expressed in yeast (Figure S1a) and no unspecific binding with the isolated AD or BD domains was observed (Figure S2). Among the single mutants, the A1020E substitution drastically reduced RGA5_Cter_ self‐interaction. Indeed, while yeast expressing the AD‐ and BD‐fused RGA5_Cter_ wild‐type constructs grew on selective media supplemented with up to 20 mM 3‐amino‐1,2,4‐triazole (3AT), those carrying the A1020E mutation grew poorly on selective medium with 1 mM 3AT and did not grow with higher 3AT concentrations (Figure 2a). The V1025Y and R1012F mutations had a weak impact on RGA5_Cter_ self‐interaction. Yeasts carrying those constructs grew on selective media containing up to 10 mM 3AT, but their growth was impaired at 20 mM 3AT (Figure 2a). Combinations of these mutations in double or triple point mutants further decreased RGA5_Cter_ self‐interaction (Figure 2b). In particular, the AE/VY and RF/AE/VY mutations almost completely abolished RGA5_Cter_ self‐interaction. The only exception was RF/AE, which showed stronger interaction than A1020E but weaker interaction than R1012F.

**FIGURE 3:**
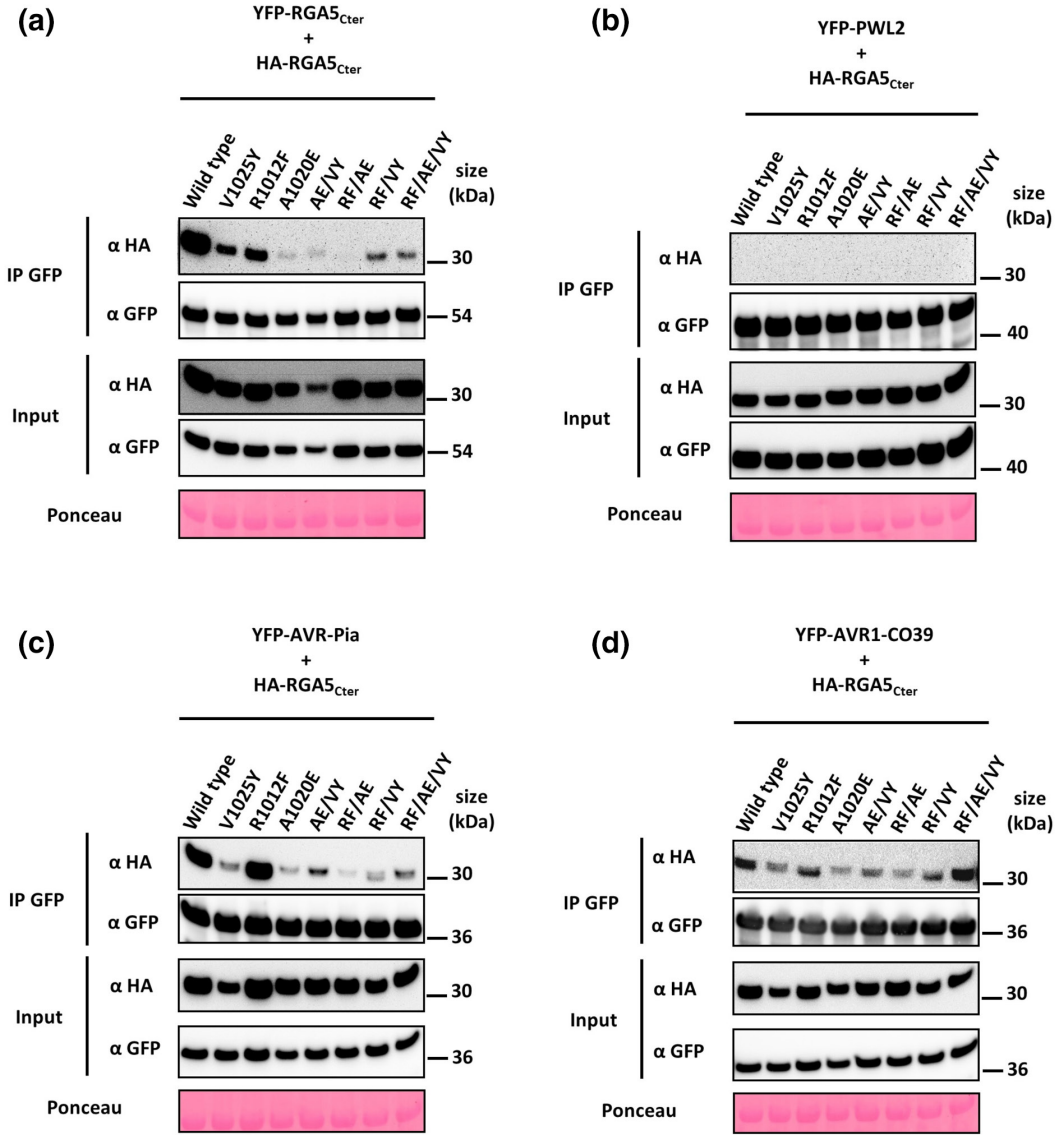
Mutations in the binding surface of RGA5_HMA_ affect RGA5_Cter_ self‐association and effector binding in coimmunoprecipitation assays. The indicated HA‐tagged RGA5_Cter_ constructs (wild type or mutants) were transiently coexpressed in *Nicotiana benthamiana* leaves with (a) the corresponding YFP‐fused RGA5_Cter_ (wild type or mutants), (b) PWL2, (c) AVR‐Pia or (d) AVR1‐CO39. Proteins were extracted after 2 days, separated by gel electrophoresis, and tagged proteins were detected in the extract (input) and after immunoprecipitation with anti‐GFP beads (IP GFP) by immunoblotting with anti‐HA (α‐HA) and anti‐GFP (α‐GFP) antibodies. Protein loading in the input is shown by Ponceau S staining of the large RuBisCO subunit. The experiments were carried out twice with similar results.

To further investigate RGA5_HMA_ self‐interaction, we performed co‐IP assays with YFP‐ and HA‐tagged RGA5_Cter_ mutants expressed in *Nicotiana benthamiana* leaves. As previously observed, YFP:RGA5_Cter_ efficiently co‐precipitated HA:RGA5_Cter_, which demonstrates self‐association of RGA5_Cter_ in planta (Figure 3a) (Guo et al., 2018). This self‐interaction was reduced by the V1025Y and R1012F mutations and almost completely abolished with the A1020E single and double point mutants. The RF/VY and the RF/AE/VY point mutants showed a weak self‐association, which was stronger than that of the A1020E single and double point mutants. All co‐IPs were specific because they were not observed with a YFP fusion of the *M. oryzae* effector protein PWL2 that does not interact with RGA5_HMA_ (Figure 3b) (Ortiz et al., 2017). Taken together, Y2H and co‐IP experiments support the important role of the selected residues in RGA5_HMA_ self‐interaction and the structural model of RGA5_HMA_ homodimer formation. In particular, the A1020E single and the AE/VY double point mutant strongly decrease self‐association in both assays and therefore appear particularly suited to address the role of HMA self‐interaction in the function of RGA5.

### 2.3 RGA5_HMA_ self‐interaction is not required for the repression of RGA4

**FIGURE 4:**
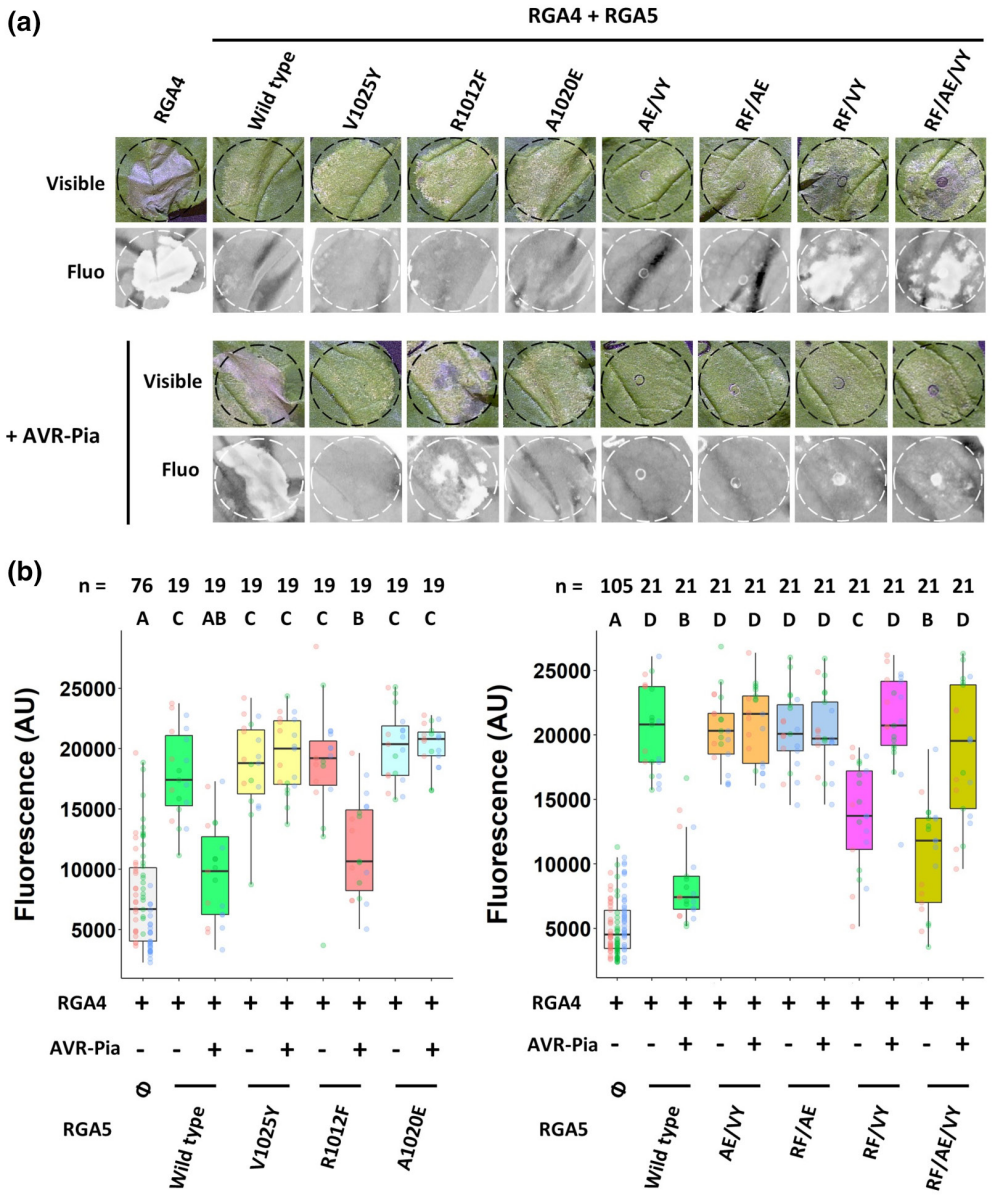
RGA5_HMA_ self‐interaction is not required for the repression of RGA4 but its interaction with AVR‐Pia is important for effector recognition in planta. (a) The *RGA4:HA* construct was transiently expressed alone or together with YFP‐tagged full‐length RGA5 constructs (wild type or variants) in the presence or absence of AVR‐Pia (without signal peptide). Pictures of representative leaves were taken 5 days after infiltration under visible light (visible) and using a fluorescence scanner (fluo) to measure a drop in red autofluorescence indicating cell death. (b) Quantification of immune responses induced in *Nicotiana benthamiana* leaves by the transient expression of constructs described in (a) (left panel: single point mutants; right panel: double and triple point mutants). Quantification was performed as described by Xi et al. (2021). Leaves were imaged 5 days after infiltration using a fluorescence scanner with a 635 nm laser used for excitation and an LPR filter (λ ≥ 665 nm) to collect the red fluorescence. Images were processed using ImageJ to measure the red fluorescence by quantifying the mean grey value for each infiltrated area (expressed as an arbitrary unit, AU). Data were plotted and dots with different colours (red, green, and blue) correspond to three biological replicates. The total number of replicates (*n*) for each condition is indicated. The boxes represent the first quartile, median, and third quartile. Difference of red fluorescence levels was assessed by a one‐way analysis of variance followed by a Tukey HSD test. Groups with the same letter (A to D) are not statistically different at level 0.05.

To determine whether the self‐interaction of RGA5_HMA_ is relevant for the function of RGA5 and, in particular, its ability to repress RGA4, we performed cell death assays in *N. benthamiana*. The previously analysed point mutations were introduced individually or in combination into full‐length RGA5 tagged N‐terminally with YFP. These mutant proteins were coexpressed with RGA4:HA by *Agrobacterium tumefaciens‐*mediated transient expression in *N. benthamiana* leaves. Immunoblotting analysis demonstrated that RGA5 mutant proteins were expressed properly (Figure S1b). Qualitative and quantitative analysis of cell death in the infiltration zones showed that all single mutants were not altered in their ability to repress RGA4‐triggered cell death when compared to RGA5 wild‐type (Figure 4a,b). Among the point mutant combinations, RGA5_RF/AE and RGA5_AE/VY were also as active as the wild type in repressing RGA4 and only RGA5_RF/VY and RGA5_RF/AE/VY showed a significantly reduced but still detectable RGA4 repressor activity. It therefore appears that in the investigated point mutants the effect on HMA self‐interaction is not correlated with the effect on RGA4 repressor activity. In particular, the A1020E single and the AE/VY double point mutant consistently impaire HMA self‐interaction in Y2H and co‐IP assays but do not influence the ability of RGA5 to repress RGA4. Reduced RGA4 repression in the case of RF/VY and the triple point mutant seems rather a consequence of the specific combination of these two point mutations, which on their own do not influence RGA4 repression and only slightly HMA self‐interaction.

Taken together, these results indicate that the self‐interaction of the HMA domain of RGA5 is not required for the repression of RGA4.

### 2.4 Mutations in RGA5_HMA_ self‐interaction and effector‐binding surface impair AVR1‐CO39 and AVR‐Pia binding

Previous structure–function analyses had demonstrated that the AVR1‐CO39 and AVR‐Pia surfaces, which interact with RGA5_HMA_, are essential for the recognition of these effectors (Guo et al., 2018; Ortiz et al., 2017). However, the role of the RGA5_HMA_ effector‐binding surface in the recognition of AVR1‐CO39 or AVR‐Pia has not been verified. To investigate this, we assessed whether the point mutations in the self‐interaction and effector‐binding surface of RGA5_HMA_ affect interaction with AVR1‐CO39 and AVR‐Pia.

**FIGURE 5:**
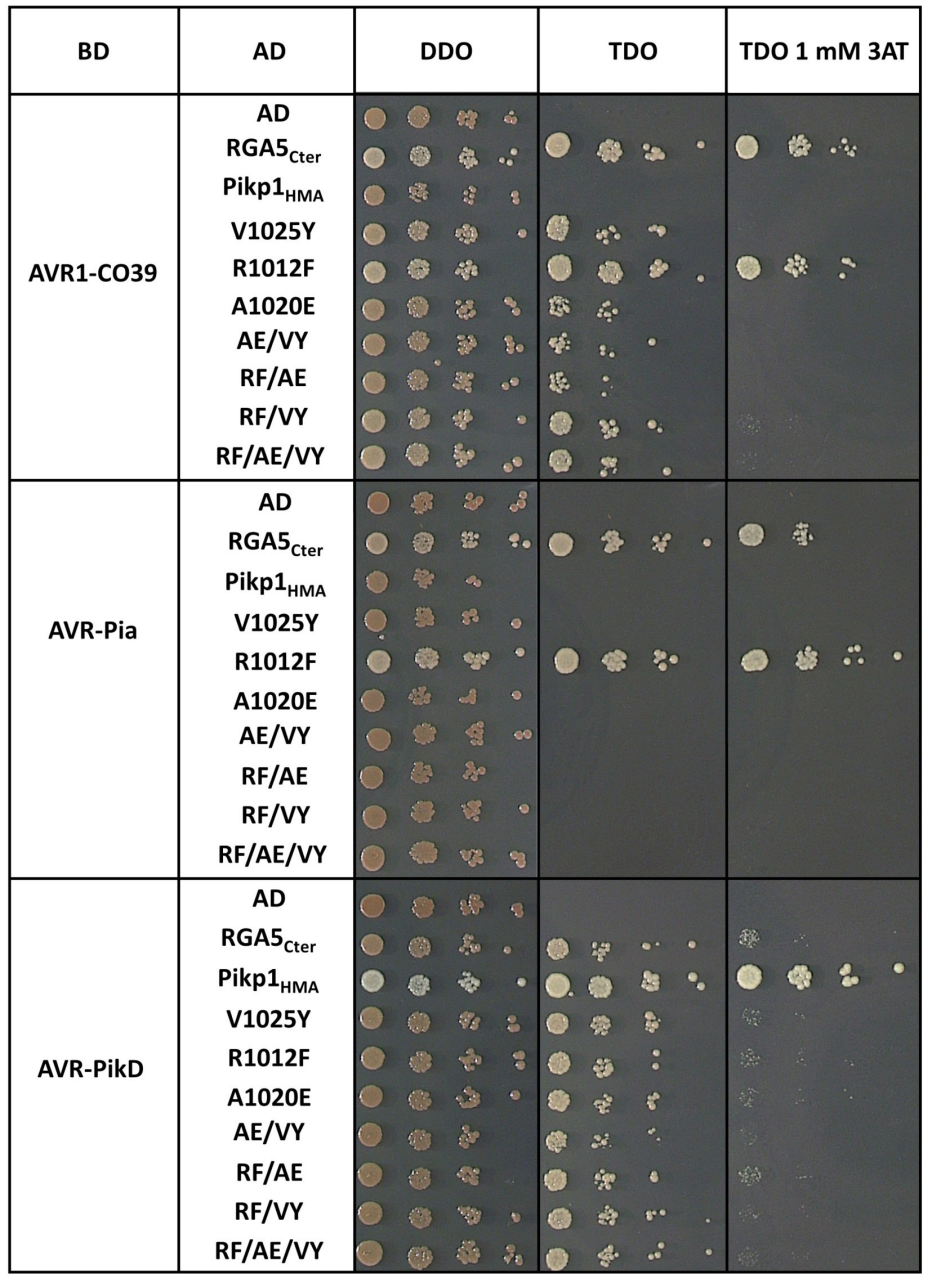
Mutations in the binding surface of RGA5_HMA_ affect RGA5_Cter_ interaction with AVR1‐CO39 and AVR‐Pia in yeast two‐hybrid assays. The interaction between the indicated BD‐fused effectors (deleted for their signal peptides) and AD:RGA5_Cter_ wild type (residues 883 to 1116) or variants carrying point mutations in the HMA domain was assayed by yeast two‐hybrid assay. The BD:AVR‐PikD, AD:Pikp1_HMA_, and AD constructs were used as controls. Dilution series of diploid yeast cells were spotted on synthetic triple dropout (TDO) medium (−Leu/−Trp/−His) and TDO supplemented with 1 mM 3AT to detect the interactions. Proper growth of the yeasts on synthetic double dropout (DDO; −Leu/−Trp) medium indicated successful mating. Pictures were taken after 5 days of growth and experiments were repeated twice with identical results.

First, we performed Y2H assays using BD‐fused effector proteins and the AD:RGA5_Cter_ constructs already used in the self‐interaction assays. Controls, used in addition to the isolated AD and BD domains (Figure S2), were the HMA domain of the rice NLR Pikp‐1 fused to the AD domain and a BD fusion of the *M. oryzae* effector AVR‐PikD. No interaction of BD:AVR‐Pia or BD:AVR1‐CO39 with the isolated AD domain or AD:Pikp1_HMA_ was detected (Figure 5). However, there was weak binding of BD:AVR‐PikD to AD:RGA5_Cter_ as previously reported (Cesari et al., 2013). Probably, this interaction is mediated by the RGA5_HMA_ surface that is analogous to the AVR‐Pik‐binding surface in Pikp1_HMA_ and that is on the opposite side of the AVR1‐CO39 and AVR‐Pia binding site (Guo et al., 2018). This would be consistent with the finding that this weak interaction is not altered by the point mutations in the RGA5_HMA_ domain and suggests that there are no major structural alterations in these mutant proteins (Figure 5).

Almost all RGA5_HMA_ mutants completely lost AVR‐Pia binding and were drastically impaired for interaction with AVR1‐CO39. Only the R1012F single point mutant was unaffected and bound both effectors with the same strength as the wild‐type domain (Figure 5). Proper expression of the different constructs was determined by immunoblotting (Figure S1a).

To further investigate how RGA5_Cter_ mutations affect effector binding, we performed co‐IP assays with YFP‐tagged effectors coexpressed with HA‐tagged RGA5_Cter_ variants in *N. benthamiana*. As in the Y2H assays, the R1012F mutant appeared unchanged as it was coprecipitated by both effectors as efficiently as the wild‐type domain (Figure 3c,d). All other single mutants or mutant combinations showed drastically reduced association with AVR‐Pia (Figure 3c). However, none of them caused complete abolition of AVR‐Pia association. Interaction with AVR1‐CO39 was also attenuated in all HMA mutants with the exception of the triple point mutant RF/AE/VY (Figure 3d). However, the association with AVR1‐CO39 was less affected than the association with AVR‐Pia by the RGA5_Cter_ mutations.

Taken together, mutations in the effector‐binding surface of RGA5_HMA_ identified in structural analysis affect the interaction with AVR‐Pia and AVR1‐CO39 in a similar but slightly different manner. Therefore, these experiments further confirm our current model of effector recognition by the integrated HMA domain of RGA5. They also indicate differences in the mechanisms of AVR1‐CO39 and AVR‐Pia binding that were already suggested by modelling studies but that could not yet be precisely determined, as the structure of the AVR‐Pia/RGA5_HMA_ complex has not been resolved yet.

### 2.5 HMA mutations that impair effector binding abolish AVR‐Pia recognition

To test whether the RGA5_HMA_ mutations affect effector recognition, we analysed in *N. benthamiana* agroinfiltration assays cell death induction upon coexpression of AVR‐Pia, RGA4, and RGA5 variants carrying in the HMA single point mutations or their combinations. Recognition of AVR1‐CO39 cannot be studied in *N. benthamiana* because its coexpression with RGA4 and RGA5 does not trigger cell death in this system, as previously reported (Cesari, Kanzaki, et al., 2014). Among all mutants, only RGA5_R1012F retained the activity to recognize AVR‐Pia (Figure 4a,b). Indeed, the AVR‐Pia‐triggered cell death response with this mutant was indistinguishable from that observed with wild‐type RGA5. The four RGA5 mutants that are able to repress RGA4 (A1020E, V1025Y, AE/VY, and RF/AE) proved unable to recognize AVR‐Pia as they did not support cell death upon coexpression with AVR‐Pia and RGA4. These results are consistent with the Y2H and co‐IP data that showed drastically reduced effector binding for the corresponding RGA5_Cter_ variants. Taken together, these experiments further highlight the importance of the effector‐binding surface of RGA5_HMA_ for effector recognition and fully support our current model of effector recognition by RGA5.

Unexpectedly, coexpression with AVR‐Pia restored the capacity of RGA5_RF/VY and of the triple point mutant to repress RGA4 (Figure 4a,b).

## 3 DISCUSSION

Previous work established the crucial role of the HMA domain of RGA5 in effector recognition and uncovered the underlying molecular mechanisms (Cesari et al., 2013; Cesari, Kanzaki, et al., 2014; Guo et al., 2018; Ortiz et al., 2017). NMR titration and X‐ray crystallography revealed that the MAX effectors AVR1‐CO39 and AVR‐Pia bind to the RGA5_HMA_ domain through their β‐strand 2 and residues before and at the beginning of β‐strand 1 (Guo et al., 2018; Ortiz et al., 2017). Replacement of critical residues within this β1‐β2 surface in both effectors drastically reduces or abolishes HMA‐binding in vivo and in vitro and impairs their recognition on infection of rice by *M. oryzae* (Guo et al., 2018; Ortiz et al., 2017). This surface is therefore crucial for the perception of AVR1‐CO39 and AVR‐Pia by RGA5.

The corresponding effector‐binding surface in the RGA5_HMA_ domain was also revealed by structure analyses but has not been validated yet by mutant analysis. X‐ray crystal structure analysis of the AVR1‐CO39/RGA5_HMA_ complex delimited the effector‐binding surface to α‐helix A and β‐stand 2 of RGA5_HMA_ and showed that binding largely relies on backbone contacts between the antiparallel β2 of the effectors and β2 of the HMA (Guo et al., 2018). Because this mechanism of effector/HMA binding involves few side‐chain residues, it is relatively unspecific and only of moderate affinity. Indeed, in vitro binding studies with RGA5_HMA_ and AVR1‐CO39 or AVR‐Pia found *K*_D_ values in the micromolar range (Cesari et al., 2022; Guo et al., 2018).

The integrated HMA domain of another, unrelated rice NLR, Pik‐1, plays also a crucial role in the allele‐specific recognition of its corresponding effector, AVR‐Pik from *M. oryzae*, which also belongs to the MAX effector family (de Guillen et al., 2015; Maqbool et al., 2015). AVR‐Pik recognition relies on high‐affinity binding to Pik‐1_HMA_, but the AVR‐Pik‐binding surface in Pik‐1_HMA_ is completely different from the effector‐binding surface of RGA5_HMA_ as it is formed by the β‐strands 3 and 4 (De la Concepcion et al., 2018; de Guillen et al., 2015; Maqbool et al., 2015). In addition, the underlying binding mechanism is different. This indicates a convergent evolution towards MAX effector recognition in these HMA IDs. However, interestingly, the αA‐β2 surface of the Pikp‐1 HMA domain can also bind AVR‐Pia (Varden et al., 2019). Modelling suggests that the AVR‐Pia/RGA5_HMA_ complex resembles the AVR1‐CO39/RGA5_HMA_ and AVR‐Pia/Pikp‐1_HMA_ complexes, and that binding involves a nearly identical effector/HMA interface and binding mechanisms (Figure S3) (Cesari et al., 2022). However, this has not yet been validated by structure or functional studies.

In this study, we undertook mutant analysis in RGA5_HMA_ to verify the effector‐binding surface identified by structure analysis and to test its role in effector recognition. Our results show that the single point mutations A1020E in the α‐helix A and V1025Y in the β‐strand 2 of RGA5_HMA_, as well as their combination, drastically reduced or abolished binding of AVR‐Pia and AVR1‐CO39 to the HMA domain in Y2H and in co‐IP assays. When these mutations were introduced into full‐length RGA5, they abolished effector recognition in *N. benthamiana* cell death assays. This provides strong additional support for the validity of the three‐dimensional structures of the AVR1‐CO39/RGA5_HMA_ and the AVR‐Pia/RGA5_HMA_ complexes, and indicates that binding of effectors to the HMA surface formed by α‐helix A and β‐strand 2 is crucial for effector recognition by RGA5.

We also addressed the question of whether the previously described self‐interaction of the HMA domain of RGA5 is relevant for the function of this sNLR (Guo et al., 2018). We hypothesized that HMA self‐interaction may be involved in the repression of RGA4 at the resting state, in absence of effectors. A role in activation was excluded because the self‐interaction interface formed by HMA α‐helix A and β‐strand 2 is largely identical to the effector‐binding surface and, consequently, HMA self‐interaction should not occur in the presence of effector. The point mutation A1020E and the double point mutation A1020E/V1025Y nearly disrupted or completely abolished the formation of RGA5_HMA_ homodimers in Y2H and co‐IP assays. Nevertheless, their introduction into full‐length RGA5 did not affect the repression of RGA4. This indicates that HMA self‐interaction is not required for the repression of RGA4 by RGA5.

This result is consistent with our previous finding that an HMA deletion mutant of RGA5 still represses RGA4 (Cesari, Kanzaki, et al., 2014). Therefore, there is currently no indication for the role of HMA self‐interaction in RGA5 function. Interestingly, similar conclusions were drawn for the Pikp‐1_HMA_ domain, which also forms homodimers through the αA‐β2 surface (Maqbool et al., 2015).

Whether HMA self‐interaction occurs in the context of RGA5 full‐length proteins, in particular at the resting state and in complex with RGA4, remains unknown. Determination of the stoichiometry of the RGA4/RGA5 resting state complex and analysis of its three‐dimensional structure will be critical to resolve this issue. Such studies will also provide important information on the position and function of IDs in the overall structure of sNLRs of the MIC1 clade, which is largely unexplored. Indeed, the fact that the HMA deletion mutant of RGA5 still represses RGA4 suggests that the HMA domain is not involved in maintaining RGA5 in a repressive state. It rather suggests that the HMA domain acts exclusively in effector recognition and receptor activation, that is, in the transition of RGA5 from a repressive to a nonrepressive state in the presence of effector. This is different from the role of the ID in RRS1 that is crucial for proper repression of its hNLR RPS4 (Ma et al., 2018).

To accomplish its role in receptor activation, the event of effector binding to RGA5_HMA_ must be somehow connected to the rest of RGA5. The simplest mechanism for this would be the engagement of additional interactions of the effector and/or the HMA with other sites in the RGA4/RGA5 complex on effector/HMA complex formation. Such interactions could induce overall conformational changes in RGA5 resulting in RGA4 derepression and are consistent with the finding that AVR‐Pia and AVR1‐CO39 interact with the HMA deletion mutant of RGA5 (Cesari et al., 2022; Ortiz et al., 2017).

In this context, the results obtained with the RF/VY double and RF/AE/VY triple point mutants are interesting (Figure 4a,b). These mutants no longer repress RGA4 on their own, but recover repressor activity in the presence of AVR‐Pia. A possible explanation is that the two mutant HMA domains engage intramolecular interactions in RGA5 that trap RGA5 in a conformation unable to repress RGA4. Interaction of AVR‐Pia with its hypothetical binding sites outside the HMA may disrupt these intramolecular interactions. This could occur either by competition, if in both cases the same binding sites are involved, or by conformational changes or steric effects resulting from AVR‐Pia‐binding. Because, according to Y2H and co‐IP experiments, AVR‐Pia does not bind the HMA in full‐length RGA5_RF/VY or RGA5_RF/AE/VY, interaction of the effector with its other binding sites in RGA5 would only interrupt intramolecular interactions engaged by mutant HMA and allow RGA5 to adopt the conformation that represses RGA4.

However, this multiple binding site model is challenged by a recent report on the engineering of RGA5 for recognition of AvrPib, another MAX effector from *M. oryzae* (Liu et al., 2021). This study shows that mutations in the HMA domain at residues of the AVR1‐CO39‐binding surface (αA and β2), as well as proximal C‐terminal residues, confer AvrPib binding, recognition and immunity in rice. Co‐IP data show that AvrPib does not bind to wild‐type RGA5, indicating that this effector does not associate with any domain of this NLR (Liu et al., 2021). The affinity of AvrPib to the engineered RGA5_HMA_‐C‐terminal fragment (*K*_D_ of 150 μM) is much lower than that of AVR‐Pia to the wild‐type RGA5_HMA_. This suggests that weak effector binding to the HMA domain alone is sufficient for recognition. Another recent study on the molecular engineering of RGA5 suggests that it is important that this interaction occurs at the αA‐β2 surface (Cesari et al., 2022). Indeed, introduction of a high‐affinity AVR‐PikD‐binding site in the β3‐β4 surface of RGA5_HMA_, with a *K*_D_ in the nanomolar range, seems less efficient in creating a new effector recognition specificity. Indeed, it only confers AVR‐PikD recognition in the heterologous *N. benthamiana* system but not immunity to *AVR‐PikD* isolates of *M. oryzae* in rice (Cesari et al., 2022).

Taken together, this study provides additional support for the central role of the ID in effector recognition by NLRs of the MIC1 clade and raises new questions on the function of the RGA4/RGA5 receptor complex. To achieve further molecular understanding of effector recognition and signalling activation by RGA4/RGA5, medium or high‐resolution structure analysis with full‐length NLRs will be critical.

## 4 EXPERIMENTAL PROCEDURES

### 4.1 Plant growth

*N. benthamiana* plants were grown in a growth chamber at 22°C with a 16‐h light period.

### 4.2 FoldX analysis and mutation selection

The PDB files, 5ZNE (dimer) and 5ZNG (complex), were first submitted to FoldX with the *RepairPDB* option (Guerois et al., 2002; Schymkowitz et al., 2005). Interfaces of the RGA5_HMA_ dimer and AVR1‐CO39/RGA5_HMA_ complex were analysed with the *AnalyseComplex* command that gave the lists of residues at the interface. All these interfacial residues were individually mutated (excluding Cys and Pro mutations) using the FoldX *BuildModel* command giving the changes in binding energy associated with the individual substitutions. Point mutants were selected among substitutions with strong impact on dimer formation and effector binding but weak impact on structure.

### 4.3 Constructs

All the primers used in this study are listed in Table S4. Details of constructs are provided in Table S5. ENTRY vectors carrying sequences for *RGA4* (1–996), *RGA5* (1–1116), *RGA5*_*Cter*_ (883–1116), *dSP‐AVR‐Pia* (20–85), *dSP‐AVR1‐CO39* (22–89), *dSP‐AVR‐PikD* (22–113), *dSP‐PWL2* (22–145), and *Pikp‐1*_*HMA*_ (182–263) are described elsewhere (Cesari et al., 2013; Cesari, Kanzaki, et al., 2014; Cesari et al., 2022; Ortiz et al., 2017). Other ENTRY plasmids carrying *RGA5* sequences (i.e., full‐length *RGA5* or *RGA5*_*Cter*_) with point mutations in the HMA coding sequence were generated by site‐directed mutagenesis (QuikChange Lightning; Agilent) using the above‐mentioned ENTRY clones, *RGA5* (1–1116) and *RGA5*_*Cter*_ (883–1116), as templates and appropriate primers (Tables S4 and S5). Plasmids for Y2H, cell‐death assays, and co‐IP experiments with mutations in the HMA sequence of full‐length *RGA5* and *RGA5*_*Cter*_ were generated by Gateway LR cloning (Life Technologies) using the ENTRY vectors described above and appropriate destination vectors listed in Table S5. All other expression constructs were generated and described in other studies as specified in Table S5 (Cesari et al., 2013; Cesari, Kanzaki, et al., 2014; Cesari et al., 2022; Guo et al., 2018; Ortiz et al., 2017).

### 4.4 Y2H assays

Y2H assays followed the protocol of the Matchmaker Gold yeast two‐hybrid system (Clontech) as described previously (Cesari et al., 2013). Briefly, saturated cultures of diploid yeasts were diluted (1/10, 1/100, 1/1000, and 1/10000) and spotted on synthetic double dropout (DDO) medium (lacking leucine and tryptophan) and triple dropout (TDO) medium (lacking leucine, tryptophan, and histidine) supplemented with various concentrations of 3AT. Pictures were taken after 5 days of growth at 28°C.

### 4.5 Transient protein expression in *N. benthamiana* and cell‐death assays

Agroinfiltration in *N. benthamiana* was performed as previously described (Cesari et al., 2013; Xi et al., 2021). *Agrobacterium tumefaciens* GV3101 (pMP90) strains carrying the *RGA4:HA*, *YFP‐RGA5* (wild type or variants), *AVR‐Pia* and *P19* construct were mixed and infiltrated at final OD_600_ 0.25, 0.5, 0.6, and 0.1, respectively. The *Agrobacterium* strain carrying the *YFP* construct was used to equilibrate the final concentration of agrobacteria in each mix. Infiltrated leaves were detached 5 days after agroinfiltration and scanned using a Typhoon FLA 9000 laser scanner (GE Healthcare). Quantitative analysis of cell‐death responses was performed as described (Xi et al., 2021).

### 4.6 Protein extraction, co‐IP, and western blot

For co‐IP assays, the two *A. tumefaciens* strains carrying constructs of interest were co‐infiltrated in *N. benthamiana* leaves at a final OD_600_ of 0.5 each. For each sample, three leaf discs were harvested 2 days after infiltration. Protein extraction from *N. benthamiana* leaves and co‐IP experiments was performed as described previously (Cesari et al., 2013; Guo et al., 2018) with the following modifications. A high‐stringency buffer was used for protein extraction (50 mM Tris.HCl pH 7.5, 150 mM NaCl, 1 mM EDTA, 0.5% NP‐40, 0.05% SDS, 0.25% sodium deoxycholate, 10 mM dithiothreitol [DTT], 0.5% polyvinylpolypyrrolidone [PVPP], 1% protease inhibitor [Sigma], 1 mM phenylmethylsulfonyl fluoride [PMSF], and 1 tablet of Roche protease inhibitor for 50 ml of buffer) and for the wash steps (same composition as the extraction buffer without DTT, PVPP, and protease inhibitor). For protein extraction from yeasts, total proteins from 200 μl of saturated yeast culture were extracted as described by Kushnirov (2000), but 50 μl of NuPAGE LDS sample buffer with reducing agent (Thermo Fisher) was used instead of regular Laemmli sample buffer. For immunoblotting analysis, proteins were separated by SDS‐PAGE using NuPAGE Bis‐Tris gels (Thermo Fisher). Proteins were transferred to a nitrocellulose membrane using the iBlot device and iBlot 2 transfer stacks (Life Technologies) or by wet‐transfer to a nitrocellulose membrane (Millipore) in case of large proteins such as full‐length RGA5 (wild type or mutants). All membranes were blocked in 5% skimmed milk and probed with anti‐HA‐horseradish peroxidase (HRP) (Roche, 3F10), anti‐Myc mouse monoclonal antibodies (Roche) or anti‐GFP mouse antibodies (Roche) followed, if applicable, by goat antimouse‐HRP antibodies (Sigma‐Aldrich). Labelling was detected using the SuperSignal West Femto maximum sensitivity substrate (Thermo Fisher) or the Immobilon western kit (Millipore).

## Supporting information


**FIGURE S1** Presence and integrity of protein expressed in yeasts and in *Nicotiana benthamiana*. (a) Total proteins were extracted from yeasts and proteins fused to AD or BD were detected by immunoblotting using anti‐HA or anti‐Myc antibodies, respectively. Ponceau S staining was used to check protein loading. (b) Total proteins were extracted from transiently transformed *N. benthamiana* leaves 2 days after agroinfiltration and were analysed by immunoblotting using anti‐GFP antibodies. Protein loading is shown by Ponceau S staining of the large RuBisCO subunit

**FIGURE S2** Negative controls for the yeast two‐hybrid assays. Dilution series of diploid yeast cells expressing the AD construct together with BD‐fused RGA5_Cter_ constructs (wild type or single mutants) were spotted on synthetic triple dropout (TDO) medium (−Trp/−Leu/−His) to serve as negative controls. Likewise, a dilution series of diploid yeast cells coexpressing the BD construct and AD‐fused RGA5_Cter_ constructs (wild type or single mutants) or AD:Pikp‐1_HMA_ were spotted on synthetic TDO medium. Diluted diploid yeast cells were also spotted on synthetic double dropout (DDO) medium (−Trp/−Leu) to monitor proper growth. Pictures were taken after 5 days of growth and these assays were performed twice with identical results

**FIGURE S3** Overlay of the structure of the RGA5_HMA_ in complex with AVR1‐CO39 and AVR‐Pia. RGA5_HMA_ is shown in cyan, AVR1‐CO39 in purple (according to crystal structure 5ZNG in Guo et al., 2018) and AVR‐Pia in green (according to the model in Cesari et al., 2022)

**TABLE S1** Prediction by the FoldX 4 toolkit of binding energy changes associated with individual substitution of key residues in the interaction surface of the RGA5‐HMA dimer

**TABLE S2** Alternative: Prediction by the FoldX 4 toolkit of binding energy changes associated with individual substitution of key residues in the interaction surface of the RGA5‐HMA dimer

**TABLE S3** Predicted changes in binding energies within the RGA5_HMA_ dimer and the RGA5_HMA_/AVR1‐CO39 complex induced by selected amino acid substitutions

**TABLE S4** Primers used in this study

**TABLE S5** Constructs used in this study

## Data Availability

The data that support the findings of this study are available from the corresponding authors upon reasonable request.
