# Supplementary figures and images for: The activity of the RGA5 sensor NLR from rice requires binding of its integrated HMA domain to effectors but not HMA domain self‐interaction
Source: Mol Plant Pathol. 2022 Jun 29;23(9):1320–30. doi: 10.1111/mpp.13236 (PMC9366066; doi:10.1111/mpp.13236)

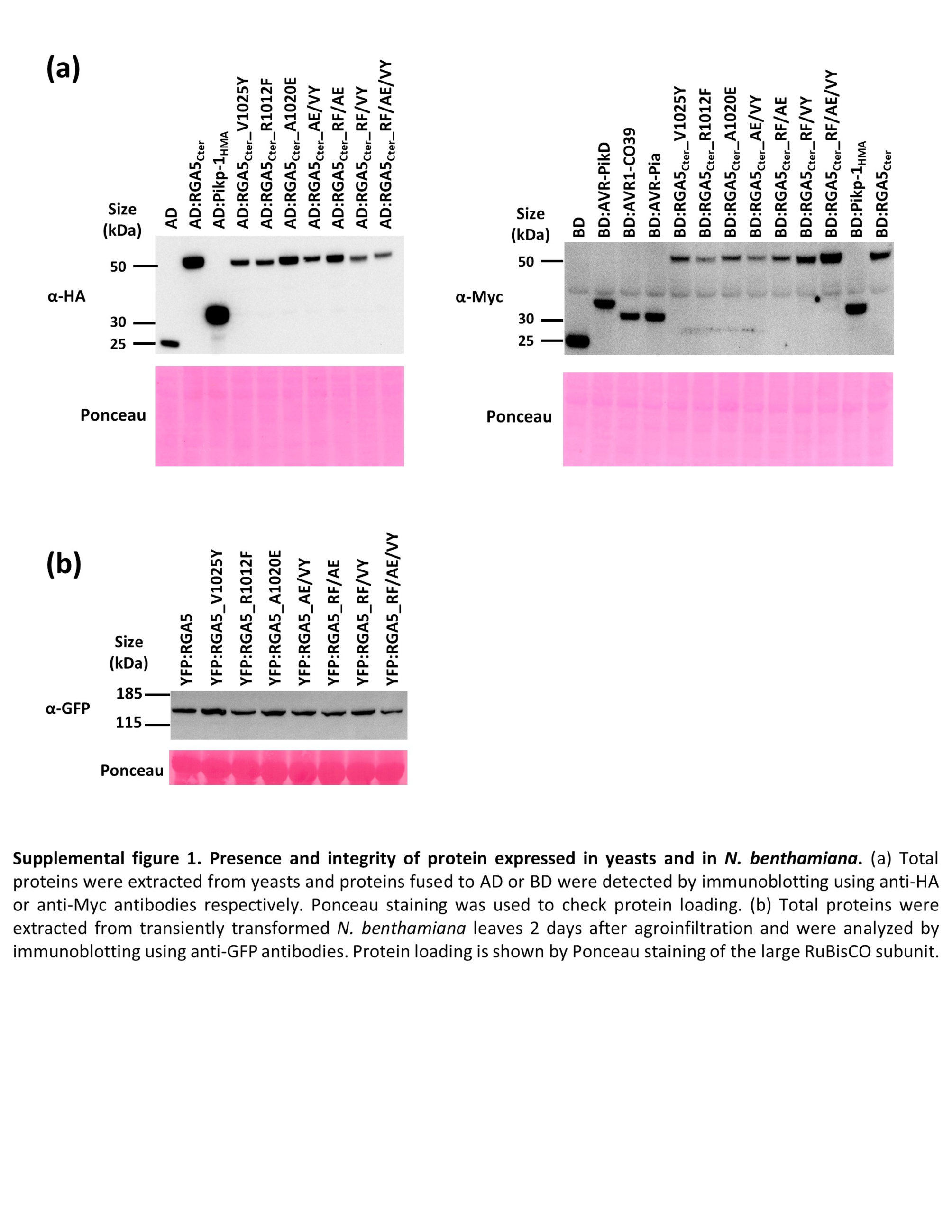

Supplement: Supplementary file 1 — FIGURE S1 Presence and integrity of protein expressed in yeasts and in Nicotiana benthamiana. (a) Total proteins were extracted from yeasts and proteins fused to AD or BD were detected by immunoblotting using anti‐HA or anti‐Myc antibodies, respectively. Ponceau S staining was used to check protein loading. (b) Total proteins were extracted from transiently transformed N. benthamiana leaves 2 days after agroinfiltration and were analysed by immunoblotting using anti‐GFP antibodies. Protein loading is shown by Ponceau S staining of the large RuBisCO subunit [file MPP-23-1320-s007.jpg]

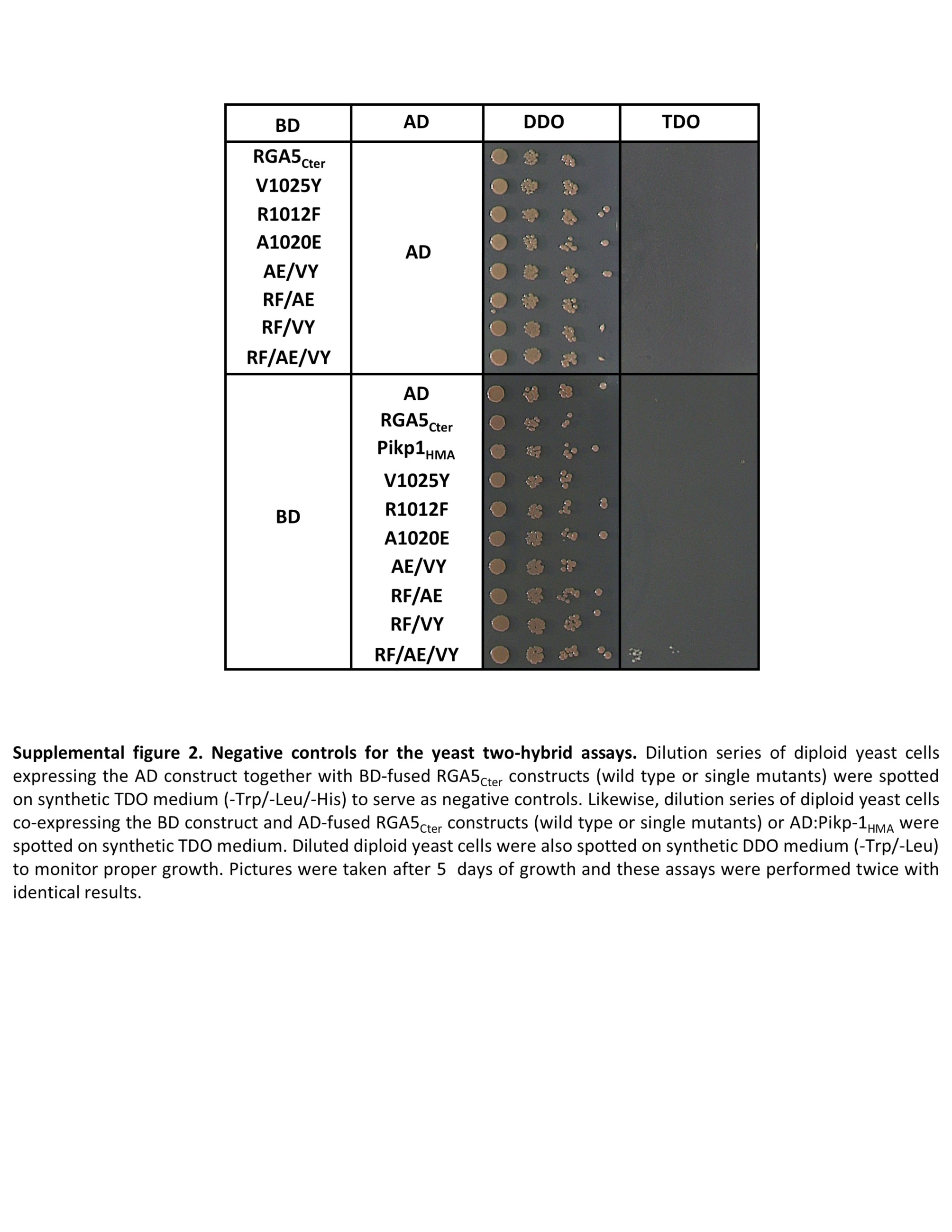

Supplement: Supplementary file 2 — FIGURE S2 Negative controls for the yeast two‐hybrid assays. Dilution series of diploid yeast cells expressing the AD construct together with BD‐fused RGA5Cter constructs (wild type or single mutants) were spotted on synthetic triple dropout (TDO) medium (−Trp/−Leu/−His) to serve as negative controls. Likewise, a dilution series of diploid yeast cells coexpressing the BD construct and AD‐fused RGA5Cter constructs (wild type or single mutants) or AD:Pikp‐1HMA were spotted on synthetic TDO medium. Diluted diploid yeast cells were also spotted on synthetic double dropout (DDO) medium (−Trp/−Leu) to monitor proper growth. Pictures were taken after 5 days of growth and these assays were performed twice with identical results [file MPP-23-1320-s006.jpg]

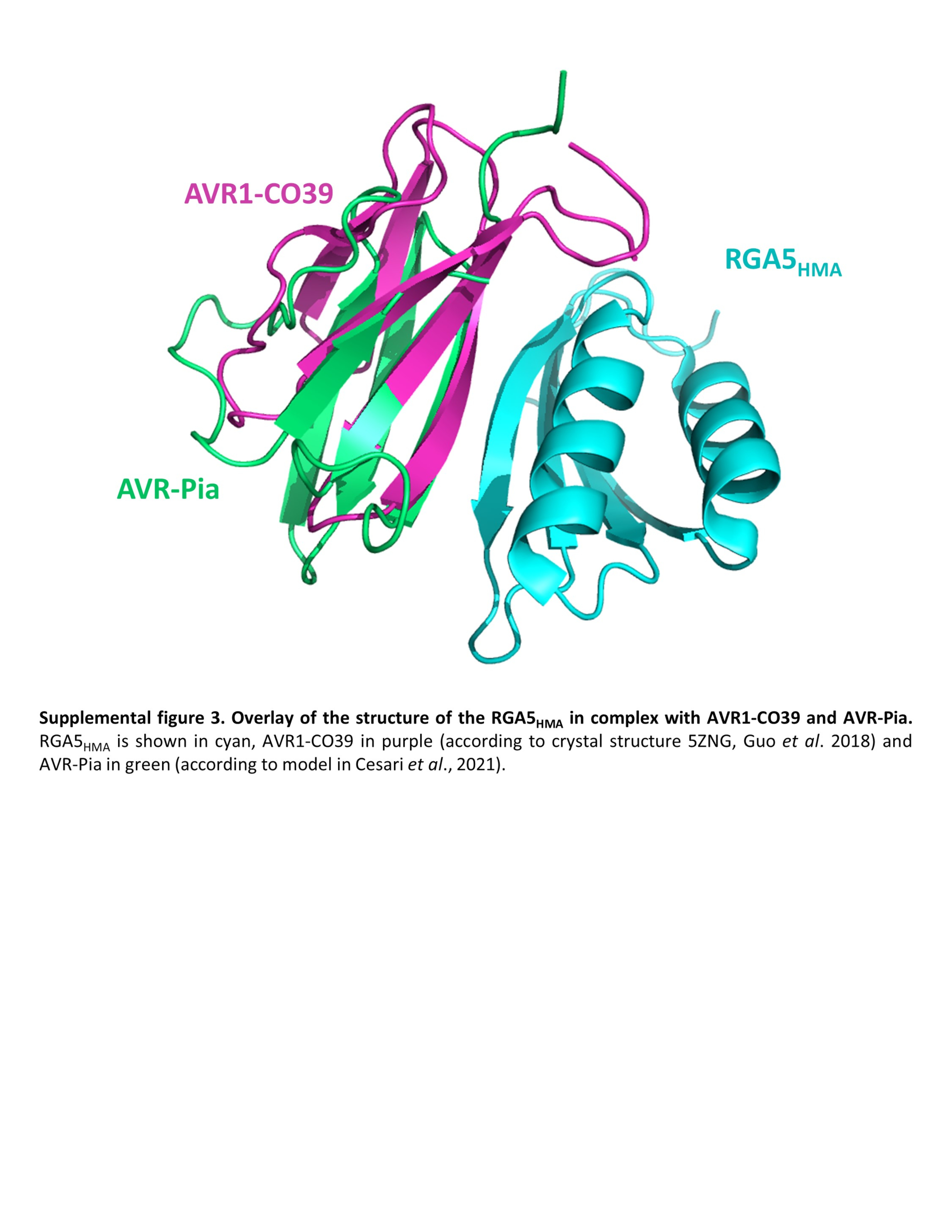

Supplement: Supplementary file 3 — FIGURE S3 Overlay of the structure of the RGA5HMA in complex with AVR1‐CO39 and AVR‐Pia. RGA5HMA is shown in cyan, AVR1‐CO39 in purple (according to crystal structure 5ZNG in Guo et al., 2018) and AVR‐Pia in green (according to the model in Cesari et al., 2022) [file MPP-23-1320-s002.jpg]
